# L*oci* identified through genome-wide association studies and lung cancer risk: is there anything more?

**DOI:** 10.1590/S1516-31802013000100026

**Published:** 2013-04-01

**Authors:** Ramon Andrade De Mello, António Araújo, Venceslau Hespanhol, Rui Manuel Reis

**Affiliations:** I MD, PhD. Medical Oncology resident, Department of Medical Oncology, Portuguese Oncology Institute; and Assistant Professor at School of Medicine, University of Porto, Porto, Portugal.; II MD, PhD. Medical Oncology Specialist, Department of Medical Oncology, Portuguese Oncology Institute, Porto, Portugal.; III MD, PhD. Pulmonology Specialist, Department of Pulmonology, São João Hospital Center; and Professor at School of Medicine, University of Porto, Porto, Portugal.; IV PhD. Senior Principal Investigator and Professor at Life and Health Sciences Research Institute (ICVS), School of Health Sciences, University of Minho, Campus de Gualtar, Braga, Portugal; Molecular Oncology Research Center, Barretos Cancer Hospital, Barretos, São Paulo, Brazil.

Dear Editor,

Recently, lung cancer screening has acquired a position of central interest among the scientific community. Many risk factors for non-small-cell lung cancer (NSCLC) susceptibility have already been identified, such as tobacco exposure, occupational exposure, random exposure, silicosis and passive smoking.^1^^,^^2^ This knowledge has raised certain questions: Is there any clinical tool suitable for screening for NSCLC with good cost-effectiveness? Is there any therapeutic choice that could improve NSCLC case prognoses through an early approach? Is there any specific population that benefit from NSCLC screening and strategies for implementing an early approach?

In 2009, a genome-wide association study (GWAS) was conducted on more than 5,000 lung cancer cases.^3^ It was reported that some genetic polymorphisms located on chromosomes 5p15.33 and 15q25.1 were associated with a risk of adenocarcinoma in a Western population.^3^ In May 2012, Ito et al.^4^ assessed the genotyping in 716 Japanese lung cancer cases and 716 matched controls. This Japanese study showed that the variants rs12914385 and rs931794 on 15q25 modified the effect of cumulative tobacco smoking on lung cancer risk, but that these two loci showed no statistically significant main effects on lung cancer risk. Furthermore, they reported that the associations shown by the TERT-CLPM1L locus on 5p15 with lung cancer risk among Japanese patients were of a similar magnitude to those among Western patients.^4^ Meanwhile, a Portuguese study^5^ reported that epidermal growth factor (*EGF*)+61 genetic A/G polymorphisms were also associated with increased risk of NSCLC in a Portuguese population. *EGF* +61 A/G genetic polymorphisms are located on chromosome 4 and have also previously been reported to be risk factors for glioma and predictive biomarkers for cetuximab in colorectal cancer cases.^5^

In this manner, the genome has now acquired a central role in NSCLC susceptibility.^3^^,^^4^^,^^5^^,^^6^ Recent studies have shown that low-dose computed tomography could serve as an important screening tool in selected populations.^5^ Currently, many therapeutic options are possible for NSCLC treatment: surgery for the early stages, and also target therapies for advanced cases with good performance status.^2^^,^^7^ We believe that taking genome studies into consideration in identifying populations at risk could improve NSCLC screening strategies and therefore would help oncologists to implement prompt approaches.
